# Deep learning approach for cancer subtype classification using high-dimensional gene expression data

**DOI:** 10.1186/s12859-022-04980-9

**Published:** 2022-10-17

**Authors:** Jiquan Shen, Jiawei Shi, Junwei Luo, Haixia Zhai, Xiaoyan Liu, Zhengjiang Wu, Chaokun Yan, Huimin Luo

**Affiliations:** 1 School of Software, Henan Polytechnic University, Jiaozuo, 454003 China; 2 School of Computer and Information Engineering, Henan University, Kaifeng, 475001 China

**Keywords:** Cancer subtype, Classification, Deep learning

## Abstract

**Motivation:**

Studies have shown that classifying cancer subtypes can provide valuable information for a range of cancer research, from aetiology and tumour biology to prognosis and personalized treatment. Current methods usually adopt gene expression data to perform cancer subtype classification. However, cancer samples are scarce, and the high-dimensional features of their gene expression data are too sparse to allow most methods to achieve desirable classification results.

**Results:**

In this paper, we propose a deep learning approach by combining a convolutional neural network (CNN) and bidirectional gated recurrent unit (BiGRU): our approach, DCGN, aims to achieve nonlinear dimensionality reduction and learn features to eliminate irrelevant factors in gene expression data. Specifically, DCGN first uses the synthetic minority oversampling technique algorithm to equalize data. The CNN can handle high-dimensional data without stress and extract important local features, and the BiGRU can analyse deep features and retain their important information; the DCGN captures key features by combining both neural networks to overcome the challenges of small sample sizes and sparse, high-dimensional features. In the experiments, we compared the DCGN to seven other cancer subtype classification methods using breast and bladder cancer gene expression datasets. The experimental results show that the DCGN performs better than the other seven methods and can provide more satisfactory classification results.

**Supplementary Information:**

The online version contains supplementary material available at 10.1186/s12859-022-04980-9.

## Introduction

### Background

Human cancer is a heterogeneous disease triggered by random somatic mutations and driven by multiple genomic alterations due to uncontrolled abnormal cell proliferation and spreading to other cells and tissues [1, 2]; cancer disrupts the intracellular homeostasis of an individual and thus seriously threatens the lives of humans. To shift to personalized treatment plans, cancers in specific tissues can be classified into subtypes based on the molecular characteristics of the primary tumour. These subtypes are the key basis for providing personalized and precise treatment [3, 4] to cancer patients and have important implications for aetiology, tumour biology and prognoses in a range of cancer research.

Gene expression data reflect the direct or indirect measurement of the abundance of gene transcript mRNA in cells. These data can be used to analyse which gene expression characteristics have changed, the correlations among genes, and how the activities of genes are affected under varying conditions. These data thus have important applications in medical clinical diagnoses, drug efficacy judgements, and in revealing disease mechanisms. Hence, gene expression data can be used in cancer subtype classification research, and many methods based on gene expression data have been presented.

### Related work

The cancer subtype classification task can be formulated as a supervised learning problem in which established tumour subtypes are used as category labels to ensure that the features learned by the model are relevant to prior biological knowledge [5]. Early research on cancer subtype classification methods usually used traditional machine learning methods. In recent years, deep learning methods have been widely used in many fields with good results, and many deep learning models are now applied in this field. Current methods can be divided into two categories.

#### Methods based on traditional machine learning approaches

In 2017, Soh et al. [6] developed tumour classification models based on random forests, logistic regression and support vector machines (SVMs) [7]. The data they used included somatic mutations, copy number variants and a combination of both, and their methods achieved a 77.7% accuracy with only 50 features. Ye et al. [8], in 2018, proposed a classification method using gene expression data based on the artificial bee colony (ABC) algorithm [9] and SVM. They used the ABC algorithm to optimize the kernel function parameters and penalty factors of SVM and obtained a relatively high classification accuracy by comparing and analysing other classification methods on a public dataset. In 2021, Duan et al. [10] used an extreme random tree model as a classifier to reduce the dimensionality of gene expression data using linear discriminant analysis, thus effectively improving the classification accuracy compared to several ensemble algorithms.

#### Methods based on deep learning approaches

In 2019, Yang et al. [11] used a stacked autoencoder (SAE) [12] neural network to learn high-level representations of gene expression data and transcriptome selective splicing data separately and then integrated all these learned high-level representations to classify patients into different cancer subtype groups, thus providing an effective and useful method for integrating multiple types of transcriptomics data to identify cancer subtypes. Zhuang et al. [13] proposed a method based on a combination of SAE and boosting to classify gene expression data in 2020. After detrending the gene expression data by principal component analysis, the SAE was used as a base classification algorithm for learning and training using boosting. Finally, multiple SAEs were combined for decision making. The authors found that the classification accuracy was improved by 5% to 10% over the use of SAE alone, exhibiting a good classification performance. Xiao et al. [14] proposed a deep learning model based on the Wasserstein generative adversarial network for unbalanced gene expression data in the same year by increasing the sample sizes in a few categories to achieve balance and further expanding the samples to improve the model performance. Majumder et al. [15] considered a combination of three deep learning models (the multilayer perceptron (MLP) and two convolutional networks) and two feature selection methods in 2022 and performed experimental analyses on four cancer datasets, achieving good performance.

In past studies applying the above methods, different measures have been taken to address the characteristics of cancer gene expression data through either linear dimensionality reductions or data equalizations. However, linear dimensionality reduction methods are easily affected by irrelevant information, some methods cannot handle high-dimensional gene table data well, and the classification results are thus not ideal [5]. To address these existing problems, we first used the synthetic minority oversampling technique (SMOTE) [16] algorithm to equalize samples. Cancer datasets are usually small, and the numbers of samples representing different categories can vary greatly. For example, in the breast cancer (BRCA) dataset used in this paper, there are only 150 samples in the sixth category, 202 in the fifth category, and 721 in the third category. Thus, we proposed a method, DCGN, by combining a convolutional neural network (CNN) [17] and bidirectional gated recurrent unit (BiGRU) [18] to achieve nonlinear dimensionality reduction in the process of learning important features. To extract key features from high-dimensional and sparse gene expression data, a relatively complex neural network is generally needed. CNNs have special structures with local weight sharing; the neuron weights (convolution kernels) on the same feature map are shared, so the neural network can learn in parallel, thus effectively reducing the complexity of the network [19]. However, a simple CNN network is prone to losing some important features during the learning process. A BiGRU can bidirectionally analyse the feature matrix of a CNN in the middle of the neural network and retain the information that may be lost through the update gate. Moreover, compared to other networks, BiGRUs are more efficient and have fewer parameters, thus expanding the model while improving its efficiency [20, 21]. Such a combined network has low complexity, is mutually complementary, and can capture comprehensive and effective features.

The main contributions can be summarized as follows. (1) By combining CNN and BiGRU, we proposed a deep learning method named DCGN that can obtain more credible features from high-dimensional sparse gene expression data. The experimental results prove that the DCGN method can obtain ideal classification results. (2) The Gaussian error linear unit (gelu) activation function is applied to the cancer classification task, and we prove through experiments that its performance is superior to those of the rectified linear unit (relu), exponential linear unit (elu), etc. (3). The DCGN performs well when applied to five high-dimensional datasets and has a good generalization ability.

## Methods

**Fig. 1:**
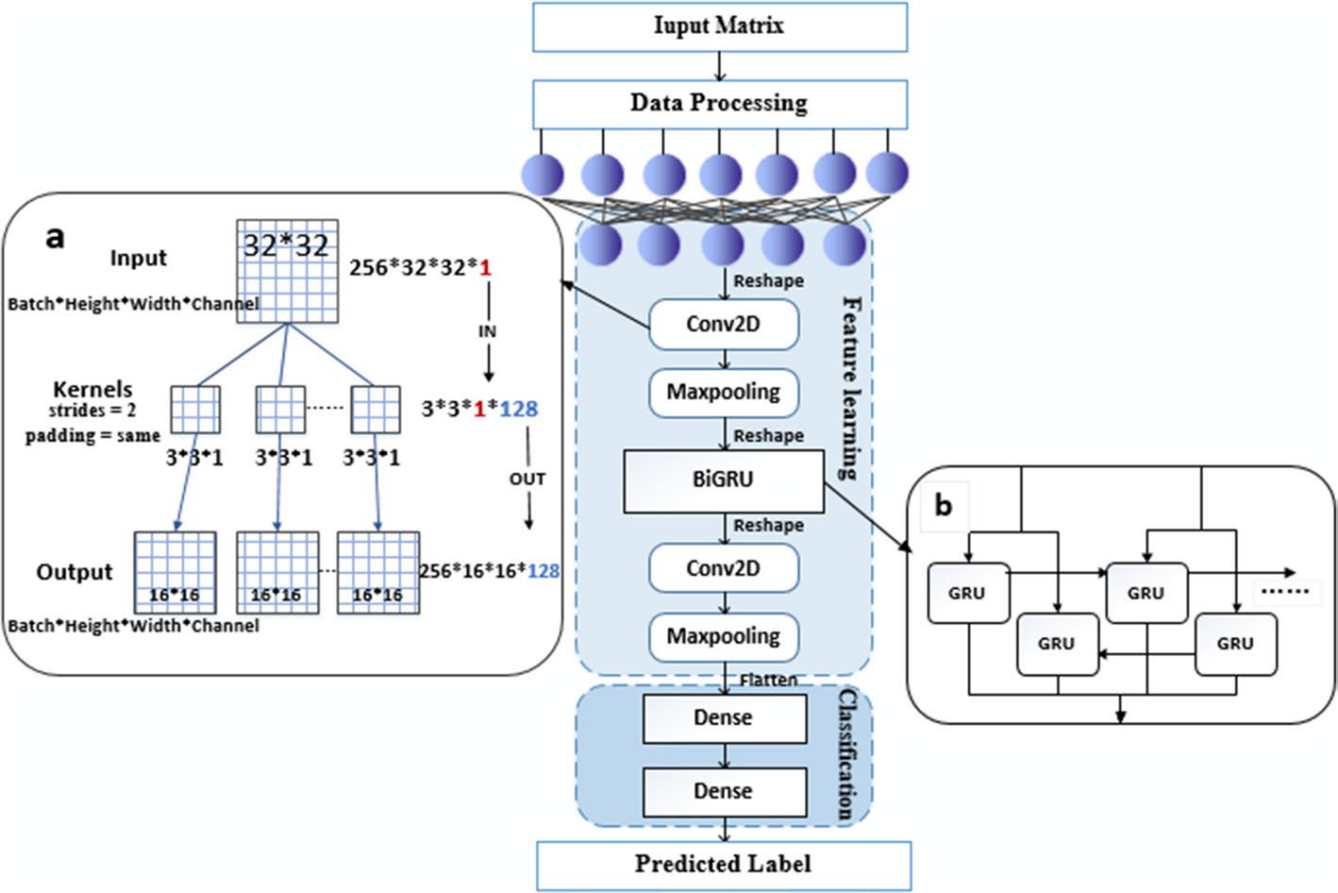
DCGN architecture

DCGN adopts gene expression data to perform cancer subtype classification. It consists of three modules (see Fig. 1). 1. The data-processing module: first, the data are enhanced by the SMOTE algorithm to solve the sample imbalance problem. Then, feature normalization is performed. 2. The feature-learning module: the most important part of the neural network. This module captures key features in the gene expression data during training. 3. The classification module: this module performs multiclassification predictions using the feature learning module outputs and compares them with the true labels to calculate the classification loss.

### Data processing module

First, considering the problem of small and unbalanced cancer samples, in this paper, we uses the SMOTE algorithm to enhance the utilized cancer datasets. The basic idea of the SMOTE algorithm is to analyseanalyze the minority class samples, artificially synthesize new samples according to the minority class samples and add them these new samples to the datasetdata set. Comparative experimental results of random undersampling and SMOTE algorithm results are provided in the Additional file 1: Table S1. The algorithm flow of SMOTE is described as follows:

1. First, classes for which the sample size is less than 15% of the total sample size are specified as minority sample sets (${\mathrm{S}}_{\mathrm{min}}$). For each sample ${x}_{i}=({d}_{1},{d}_{2}\dots {d}_{m})$ in a class with few samples (${\mathrm{S}}_{\mathrm{min}}$), m represents the dimension of the sample. We calculate the Euclidean distance to all samples in the minority class sample set ${\mathrm{S}}_{\mathrm{min}}$ and obtain the K-nearest neighbours.
2. Then, we determine the sampling multiplier N according to the sample imbalance ratio. For each minority class sample ${x}_{i}$, a number of samples ${X}_{n}$ are randomly selected from its K nearest neighbours.
3. For each randomly selected nearest neighbour ${x}_{n}$, a new sample is constructed separately from the original sample according to Eq. (1), where ${x}_{i}$ represents a sample point from the minority class and ${x}_{n}$ represents a sample point randomly selected from the K nearest neighbours. The term rand(0,1) represents a randomly generated number between 0 and 1.
  1 $$x_{new} = x_{i} + \left( {x_{n} - x_{i} } \right){*}rand\left( {0,1} \right)$$

Next is the feature normalization step. Feature normalization is performed on the gene expression data before model training so that all features in the dataset have means of zero and unit variance. Specifically, assuming that the mean of a feature is u and its standard deviation is $\upsigma$, the utilized feature standardization formula is defined as follows:

2 $$X^{\prime} = \frac{x - u}{\sigma }$$

3 $${\upsigma } = \sqrt {\frac{{(x_{1} - u)^{2} + (x_{2} - u)^{2} + \ldots (x_{n} - u)^{2} }}{n}}$$

where the calculation acts on each column, x represents the feature matrix of the data, u is the mean of the data, $\upsigma$ is the standard deviation of the data, and ${X}^{^{\prime}}$ represents the data after feature normalization.

### Feature learning module

The feature-learning module includes a fully connected layer (FC layer), a convolution layer, a BiGRU layer, and another convolution layer. The input layer first goes through the FC layer, and the FC layer computation process is essentially matrix multiplication. Then, the computational result is output by the activation function. The calculation formula is expressed as follows, where x represents the input matrix, W is the weight parameter, b represents the bias, GELU is the activation function of this layer, and Y represents the nonlinear output through the activation function.

4 $${\text{Y}} = {\text{GELU}}\left( {{\text{W}}*{\text{x}} + {\text{b}}} \right)$$

#### Activation function

The activation function is the "switch" that determines whether a neural network transmits information or not; this function is crucial in neural networks. Different activation functions greatly impact the training effect of neural networks. At present, the most commonly used activation functions are the relu(Recitified Linear Unit) function, sigmoid function, elu(Exponential Linear Unit) function, etc. In this paper, an activation function called gelu(Gaussian Error Linear Unit) is selected. Some experiments have shown that gelu is superior to other activation functions, such as relu and elu, for tasks ranging from computer vision to natural language processing [22]. We conduct comparative experiments on these activation functions in “Activation function comparison experiment” section.

**Fig. 2:**
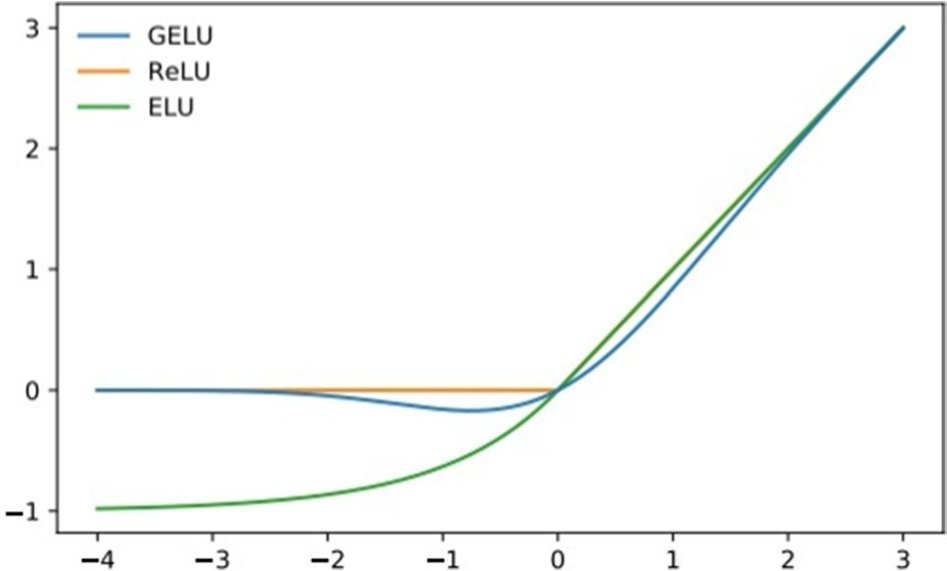
Function image of activation functions

Gelu can be seen as a combination of relu and dropout ideas. For high-dimensional gene expression data, excessive features may affect the feature-learning procedure. Sometimes we want to regularize unimportant information to zero, and the nonlinear variation feature of the gelu activation function can perform stochastic canonical transformation. It can be understood that a given input value should be multiplied by 1 or 0 according to its specific situation. For a more mathematical description, each input value x that obeys the standard normal distribution N(0,1) is multiplied by a Bernoulli distribution. In Fig. 2, for the gelu activation function, when x is larger, y is more likely to be retained, while the smaller x is, the more likely y is to be set to 0; however, but when x is less than 0, y has a certain probability not to be 0; under the relu function, outputs below 0 are set to 0. As x decreases, the probability of y being set to zero increases, and the limit is 0.

The gelu function has the following form:${\text{ GELU}}\left( {\text{X}} \right) = {\text{x}} \cdot \phi \left( {\text{x}} \right),{\text{x}}\sim{\text{N}}\left( {0,1} \right)$ (5), where x is the input, $\phi \left( {\text{x}} \right) = {\text{P}}\left( {{\text{X}} \le {\text{x}}} \right)$, X is a Gaussian random variable with zero mean and unit variance, and P(X <  = x) is the probability that X is less than or equal to a given value x. The mathematical formula applied to obtain the approximate calculation is provided in the original paper [22] as follows:

6 $${\text{GELU}}\left( {\text{x}} \right) = 0.5{\text{x}}\left( {1 + {\text{tanh}}\left( {\sqrt {2/{\uppi }} \left( {{\text{x}} + 0.044715{\text{x}}^{3} } \right)} \right)} \right)$$

#### Convolutional layer

The outputs of the FC layer are next subjected to convolution operations. Because CNN are mostly used for image processing, the calculation process typically involves a number of image channels (generally images have three channels: red, green, and blue (RGB)); however, the data used in this article are characterized by numerical matrices of gene expression data, which differ from image data, so it is necessary to describe the calculation process. The CNN calculation is different from that of the FC layer. The input matrix format includes four dimensions: the number of samples, height, width, and number of channels. The output matrix format has the same dimensional order and meaning as the input matrix, but the sizes of the last three dimensions are changed by the convolution operation. The meaning of the weight matrix (convolution kernel) dimensions is different from those of the above two matrices. These are the height of the convolution kernel, the width of the convolution kernel, the number of input channels (the number of channels in the input matrix), and the number of output channels (the number of convolution kernels).

Taking the first convolutional layer as an example, the FC layer output as (N,1024) becomes (N,32,32,1) after a Reshape operation is applied, where N represents the number of samples in the batch, the height and width are both 32, and the number of channels is only one. The first convolutional layer is the conv2D layer with 128 convolutional kernels of size (3,3,1), padding = same, strides = 2. The term "padding = same" indicates that the output size is equal to the input size divided by the strides and rounded up. The calculation process is shown in Fig. 1. Thus far, the conv2d layer has slid and multiplied the feature matrix through the convolution kernel to extract features and reduce the dimensionality. At the same time, the conv2d layer has a special structure with local weight value sharing, and this effectively reduces the training parameters of the neural network. To explore the impacts of these two convolutional layers on the DCGN, we conducted comparative experiments on different datasets, and the detailed experimental results are provided in the Additional file 1: Table S2 and Fig. S1.

The convolution layer also contains a maximum pooling layer. This layer can reduce the size of the model, improve the calculation speed, and improve the robustness of the extracted features. However, the maximum pooling layer has no parameters to learn and can only takes the maximum value from the target area; thus, the numbers of input and output channels do not change..

#### BiGRU layer

**Fig. 3:**
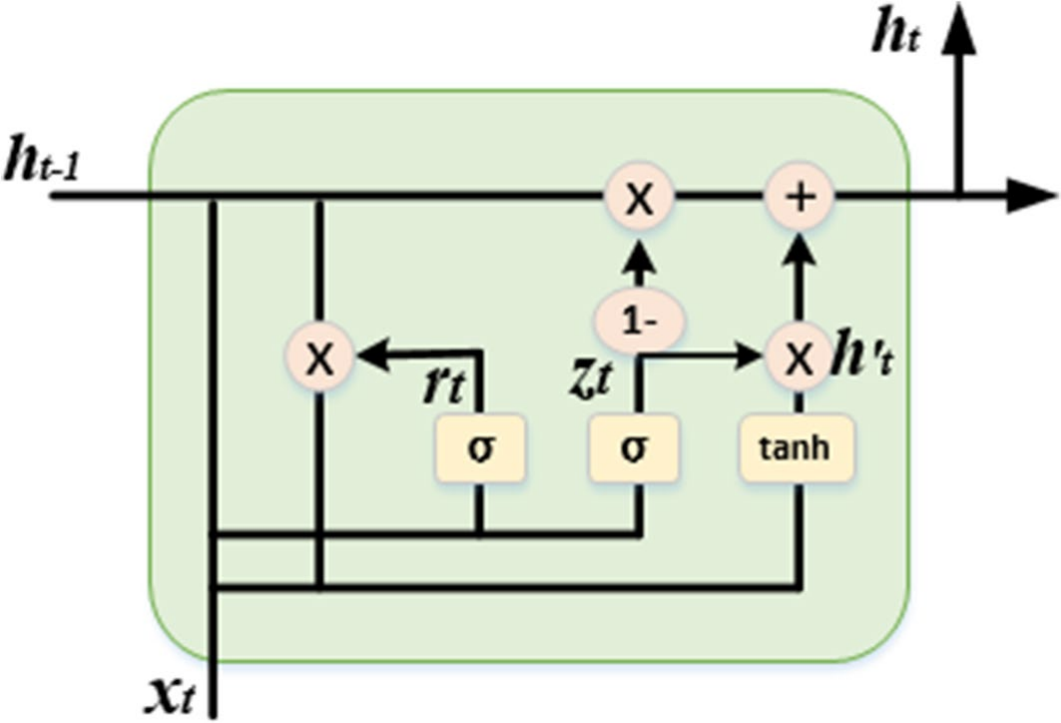
Structure of the GRU (where σ represents the sigmoid function)

The gated recurrent unit (GRU) uses an update gate and a reset gate. The update gate determines how much information from the past should be allowed to pass through the gate, while the reset gate decides how much information from the past should be discarded. First, we obtain the two gating states from the last transmission down state ${h}_{t-1}$ and the input ${x}_{t}$ of the current node. In Fig. 3, ${z}_{t}$ performs the update gate operation,${r}_{t}$ performs the reset gate operation, and the gate uses a sigmoid function to determine which values to let pass or discard.

7 $$z_{t} = \sigma \left( {W_{z} \cdot \left[ {h_{t - 1} ,x_{t} } \right]} \right)$$

8 $$r_{t} = \sigma \left( {W_{r} \cdot \left[ {h_{t - 1} ,x_{t} } \right]} \right)$$

When the gating signal is obtained, we first use reset gating to obtain the reset data $h_{t - 1} ^{\prime}$; then, we splice $h_{t - 1} ^{\prime}$ with the input $x_{t}$ and deflate the data to the range of − 1~1 by the tanh activation function.

9 $$h_{t - 1}^{^{\prime}} = h_{t - 1} *r_{t}$$

10 $$h_{t}^{^{\prime}} = {\text{tanh}}\left( {W \cdot \left[ {h_{t - 1}^{^{\prime}} ,x_{t} } \right]} \right)$$

Finally, the most critical step in GRU is the “update memory” step, in which we perform both forgetting and remembering tasks. The update gate $z_{t}$ is equivalent to the forgetting gate, and 1-$z_{t}$ is equivalent to the input gate. The current moment memory unit can be expressed as follows:

11 $${\text{ h}}_{t} = \left( {1 - z_{t} } \right)h_{t}^{^{\prime}} + z_{t} h_{t - 1}$$

where $\left( {1 - z_{t} } \right)h_{t}^{^{\prime}}$ denotes the selective memory of $h_{t}^{^{\prime}}$ containing information about the current node, retaining some important information, and $z_{t} h_{t - 1}$ denotes the selective obliviousness of the originally hidden state. The operation of this step is to forget the information of some passed-down dimensions in $h_{t - 1}$ and add some dimensional information entered by the current node.

The above process represents forward delivery, and the BiGRU layer contains both forward and reverse gated recurrent units, as shown in Fig. 1b. At each moment t, the input is provided to the gated unit in both directions, while the output is determined by the joint bidirectional output.

### Classification module

The DCGN takes the output of the feature-learning module into the classification module composed of four fully connected layers after a straightening operation is performed. We use a dropout layer after each fully connected layer to improve the generality of the network and to mitigate the interactions between neurons. The last layer directly outputs the tensor computed by the network, which may be optimally numerically stable.

#### Loss function

The loss function of the neural network is the SparseCategoricalCrossentropy function; this function can convert digital coding into a one-hot coding format and then apply the cross entropy loss function to the data (real label value) and the predicted label value. The multicategorical cross-entropy loss function is actually an extension of the bicategorical cross-entropy loss function, as expressed in Eq. (12), where M is the number of categories;${y}_{ic}$ is the symbolic function (0 or 1) (if the true class of sample i is equal to c, this term takes a value of 1; otherwise, it takes a value of 0); and ${p}_{ic}$ is the predicted probability that sample i belongs to category c.

12 $${\text{ Loss}} = - \frac{1}{{\text{N}}}\mathop \sum \limits_{{\text{i}}} \mathop \sum \limits_{{{\text{c}} = 1}}^{{\text{M}}} {\text{y}}_{{{\text{ic}}}} {\text{log}}\left( {{\text{p}}_{{{\text{ic}}}} } \right)$$

## Experiment

### Experimental data

To demonstrate the validity of the proposed method, we conducted experiments on breast and bladder cancers. The breast cancer dataset used herein was obtained from a previous Molecular Taxonomy of Breast cancer International Consortium (METABRIC) [3] study. Chen et al. [5] processed this dataset to obtain gene expression profiles from 1989 primary breast tumour samples and 144 normal breast tissue samples with Prediction Analysis of Miroarray 50 (PAM50) subtypes [4] used as classification labels. The bladder cancer dataset was derived from The Cancer Genome Atlas (TCGA) project and contains the gene expression profiles of 408 bladder cancer samples. Four currently published molecular classifications, MD Anderson (MDA) [23], TCGA [24], Curie Institute (CIT)-Curie [25], and Lund [26], have been widely used in bladder cancer classification studies. We used the R package BLCAsubtyping [27] to label each cancer sample separately according to these four classification systems. The details are provided in Table 1.

**Table 1:** Cancers and their specific subtypes

Cancer category	Classification systems	Specific typology	Genes	Number of samples
BRCA	PAM50	Basal, HER2 + , luminal A/B, normal-like, normal	20,000	4221
BLCA	MDA	Luminal, basal, p53-like	20,087	1010
	TCGA	Luminal_infiltrated, Luminal_papillary, Luminal, Neuronal, Basal_squamous	20,000	761
	CIT-Curie	MC1, MC2, MC3, MC4, MC5, MC6, MC7	20,087	909
	Lund	UroA-Prog, UroB, UroC, Uro-Inf, GU, GU-Inf, Mes-like, Ba/Sq, Sc/NE-like, Ba/Sq-Inf	20,000	1185

### Experimental Setting

The number of nodes in the feature learning module was set to 1024, 128, 64, and 64, while the number of nodes in the classification module was set to 128, 64, 32, and 10. For the model construction and performance evaluation steps, we randomly divided the data into a training set, a validation set, and a test set containing 80%, 10% and 10% of the samples, respectively. To improve the memory utilization and parallelization efficiency in the large matrix multiplication process, we set the batch size to 256. Then, we used the Adam method [28] to tune the model parameters with the learning rate set to 1e-3 and the dropout layer ratios set to 0.6 and 0.7.

### Comparative methods

To prove the robustness of the method proposed in this paper, we selected seven methods based on machine learning or deep learning approaches to conduct experiments. The following text briefly introduces the compared methods, among which gcForest [29], SAE, BiGRU, and DCNN belong to the deep learning methods.

The basic SVM model is a linear classifier with the largest interval defined on the feature space; in the gradient boost decision tree (GBDT) [30], the decision tree (DT) and gradient boosting (GB) components provide the learning strategy, and the DT model is trained with the gradient boosting strategy. In the light gradient boosting machine (LightGBM) [31], a distributed gradient boosting framework based on a decision tree algorithm provides a framework based on the GBDT algorithm to support efficient parallel training, low memory usage and high accuracy. The gcForest method, involving a deep forest model with a cascading function, applies the principles of deep neural networks to the traditional "random forest" machine learning algorithm. SAE is a deep neural network model composed of multiple layers of Sparse AutoEncoder (sparse self-encoder); in this model, the output of the previous self-encoder layer is used as the input to the subsequent self-encoder layer, and the last layer is a classifier. In BiGRU, the bidirectional GRU layer in a recurrent neural network (RNN) retains important features through various gate functions. Compared to the long short-term memory (LSTM) model, BiGRU has fewer parameters and a better effect. DCNN is a deep learning method based on CNNs that has exhibited a good performance on gene expression datasets characterizing various cancers.

### Results and analysis

#### Evaluation metrics

Evaluation metrics are critical criteria used to measure whether a method performs well when facing a given problem. For classification problems, metrics such as the accuracy, precision, recall, F1-score, and confusion matrix [32] are normally used. The accuracy rate represents the proportion of the number of correct samples predicted by the method to the total number of samples, and the precision rate refers to the proportion of samples that are actually positive among the samples that are predicted to be positive. Recall refers to the proportion of samples that are predicted to be positive among the true positive samples. The F1-score is the harmonic mean of the precision and recall scores. By running the results of each model under the same experimental settings, we can intuitively identify the strengths and weaknesses of the model classification performance. When evaluating multiclassification problems, the problem is usually decomposed into multiple 2-classification problems, i.e., n classifications can be decomposed into n 2-classifications, with one of the classes set as the positive class and the remaining classes uniformly set as the negative class in each iteration; then, various 2-classification indexes are calculated. Finally, the multiclassification evaluation indexes are averaged. Although this paper uses the SMOTE algorithm to balance the data, it properly generates only some minority samples, so different proportions of categories may occur. Therefore, when calculating the multiclass evaluation metrics, we chose the weighted average method. When calculating the precision and recall scores, the scores of each category were multiplied by the percentage of the category in the total sample to obtain summed scores. In formulas (13) and (14), L represents the number of categories, $precision_{i}$ and $recall_{i}$ represent the precision and recall rates of class i, respectively, and $w_{i}$ represents the proportion of the i-th sample in the total sample.

13 $${\text{Precision }} = { }\frac{{\mathop \sum \nolimits_{{{\text{i}} = 1}}^{{\text{L}}} {\text{precision}}_{{\text{i}}} {\text{*w}}_{{\text{i}}} }}{{\text{L}}}$$

14 $${\text{ Recall }} = { }\frac{{\mathop \sum \nolimits_{{{\text{i}} = 1}}^{{\text{L}}} {\text{recall}}_{{\text{i}}} {\text{*w}}_{{\text{i}}} }}{{\text{L}}}$$

15 $${\text{F}}1 = { }\frac{2*Precision*Recall}{{Precision + Recall}}$$

#### Activation function comparison experiment

**Fig. 4:**
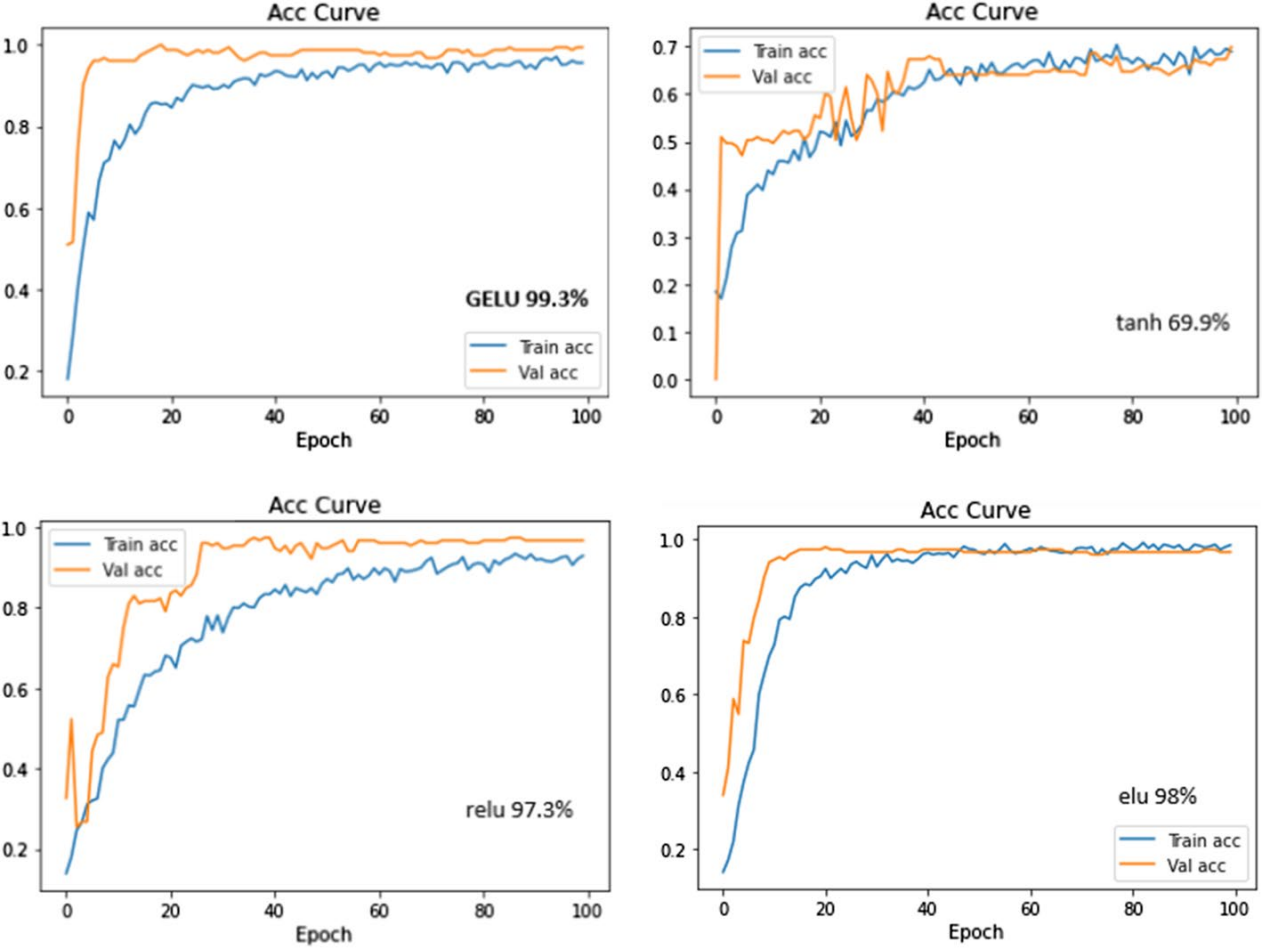
Activation functions accuracy curve (experimental results of different activation functions on other datasets are provided in the Additional file 1: Fig. S2)

To prove the influence of the gelu activation function selected in this paper on the model, we selected three other activation functions, relu, elu, and tanh, as the activation functions of the model and conducted comparative experiments on the bladder urothelial carcinoma (BLCA)-TCGA dataset. The experimental results are shown in Fig. 4. Gelu exhibited the fastest convergence speed, and its accuracy rate was as high as 99.3%. Both elu and relu performed slightly worse. Tanh had the worst training effect, with an accuracy of only 70%.

#### Ablation experiment

**Fig. 5:**
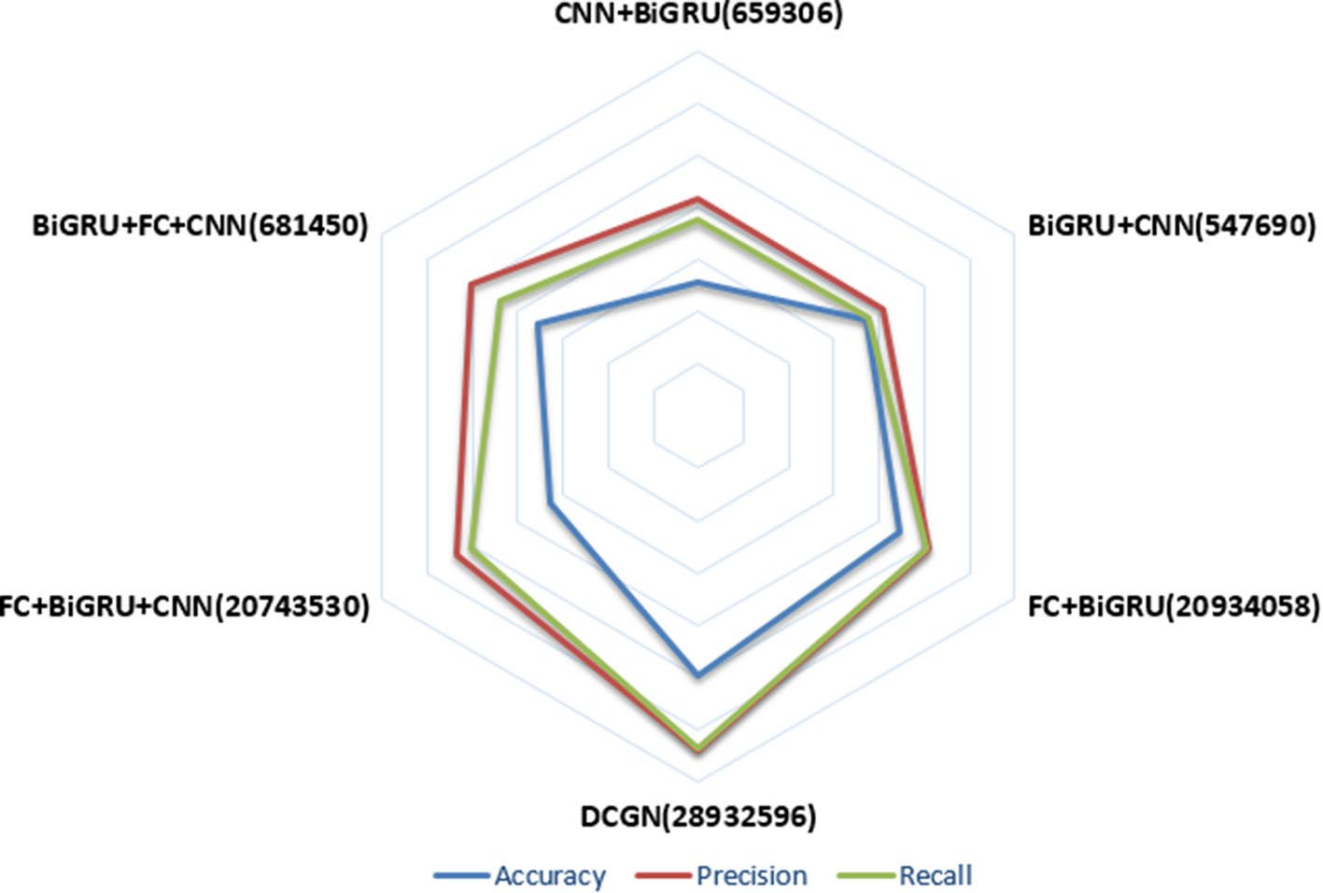
Ablation experiment results

There are many robust network structures in deep learning, and different combinations of networks may perform differently. To demonstrate that the DCGN proposed in this paper can provide better results than other network structures, we used different neural networks to obtain free combinations and verified the networks on the BRCA 20,000-dimension dataset. The experimental results are shown in Fig. 5.

From Fig. 5, it can be found that the closer the curves of the three metrics are to the edge of the hexagonal graph, the better the classification performances of the networks are. The three evaluation metrics obtained for the DCGN are very close to the edge of the hexagon, and all are higher than those of the other network models, indicating that the classification performance of the model proposed in this paper is best compared to the other analysed deep learning neural networks. In addition, the number after each network combination in the figure indicates the total number of parameters trained by the deep learning model. During the backpropagation process, each model minimizes the loss by updating the parameters corresponding to each layer. The number of model parameters has a certain relationship with the model performance. The consideration of too few parameters may affect the classification performance of a model.

#### Comparison experiments based on BRCA

To further validate the effectiveness of the proposed approach, we compared DCGN with the seven methods mentioned in “Comparative methods: section on the BRCA 20,000-dimension dataset. The dimensionality of the data matrix after data enhancement was (4221, 20,000), and the specific results are shown in Table 2.

**Table 2:** BRCA20000-dimension dataset results

*Dataset*	*BRCA*							
Methods	DCNN	SVM	GBDT	LightGBM	gcForest	SAE	BiGRU	DCGN
*Highest level*								
Accuracy	90.2	94.7	94.3	94.6	95.2	94.2	95	96
Precision	95.1	94.9	94.5	94.7	95.4	95.3	95.4	98.7
Recall	94.8	94.8	94.3	94.5	95.2	94.8	94.7	98.7
F1-score	94.6	94.8	94.3	94.6	95.3	94.7	94.8	98.6
*Average level*								
Accuracy	88.7	94	93.2	93.6	94.1	93.7	94	94.8
Precision	92.5	94	93.4	93.7	94.2	93.1	93.9	96.8
Recall	92.2	94.1	93.2	93.5	94.2	93.2	94	96.7
F1-score	92.3	94	93.2	93.6	94.3	93.2	94.1	97

From Table 2, at the highest level, it can be seen that the DCGN exceeded the other seven models in all four metrics. In particular, DCGN achieved performance improvements of ~ 3–5% in terms of the F1 scores; DCGN was the only model whose evaluation metrics exceeded 95% on average. The experimental results tentatively demonstrate that the classification performance of the DCGN is best when applied to high-dimensional datasets, indicating that the proposed method can exhibit a good classification performance on high-dimensional gene expression data. To further prove this conclusion, we verify the proposed model on the BLCA dataset.

**Fig. 6:**
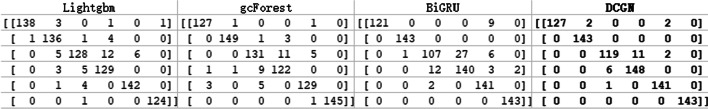
Confusion matrix derived for several well-performing methods

A confusion matrix is a situation analysis table that summarizes the prediction results of a machine learning classification model. In the form of a matrix, the situation in the dataset can be summarized according to two standards: the real category and the category predicted by the classification model. A confusion matrix thus gives a more intuitive picture of how well a model performs, because all correct predictions are shown on the diagonal and all wrong predictions are shown off the diagonal; thus, it is very straightforward to identify incorrect predictions. Figure 6 shows the confusion matrix obtained for several models with relatively high classification performances on the BRCA 20,000-dimension dataset. It can be clearly seen that the DCGN has the fewest prediction results outside the diagonal line, with only 24 samples, while the fewest number of predictions outside the diagonal among the other models is 42. The result equally proves that the classification performance of the DCGN is optimal on the BRCA 20,000-dimensional dataset.

In addition, two multiclassification metrics, the Kappa coefficient [33] and Hamming distance [34], can also reflect the classification performance of a model to some extent. The Kappa coefficient is a model evaluation parameter obtained based on the calculation of the confusion matrix with the following equation:

16 $${\text{ Kappa }} = { }\frac{{{\text{P}}_{0} - {\text{P}}_{{\text{e}}} }}{{1 - {\text{P}}_{{\text{e}}} }}$$

where ${\mathrm{P}}_{0}$ represents the overall classification accuracy and ${\mathrm{P}}_{\mathrm{e}}$ denotes the number of true samples in each category multiplied by the number of predicted samples in each category and then divided by the square of the total number of samples. The closer the Kappa coefficient is to 1, the better the classification performance is. The Hamming distance is also applicable to multiclassification problems and is simply a measure of the distance between the predicted label and the true label; this term takes a value between 0 and 1. A distance of 0 means that the predicted result is exactly the same as the true result, while a distance of 1 indicates that the model is exactly the opposite of the desired result. The experimental results are shown in Table 3. The Kappa coefficient of the DCGN reaches 0.984, indicating that the prediction results are very close, almost identical, to the actual classification results. Moreover, the Hamming distance of the DCGN is also the smallest, at only 0.013, the same as the expected experimental results. These results are strong proof that the DCGN has the best classification effect on high-dimensional datasets.

**Table 3:** Kappa coefficient and Hamming distance values of each model on the BRCA 20,000-dimension dataset

Methods	DCNN	SVM	GBDT	LightGBM	gcForest	SAE	BiGRU	DCGN
Kappa	0.937	0.947	0.937	0.937	0.936	0.953	0.937	0.984
Hamming distance	0.051	0.043	0.052	0.052	0.053	0.039	0.051	0.013

#### Comparison experiments based on the BLCA

As is widely known, the generalization ability of a model is an important criterion for judging its quality. Our proposed model performed well on the BRCA 20,000-dimension dataset. To demonstrate the generalization ability of the proposed model, we next conducted experiments on four high-dimensional BLCA datasets. The BLCA datasets were processed separately according to the four molecular typing systems as described in “Experimental data” section. After data enhancement, we loaded the data matrix and labels into each model to perform the experiments.

The experimental results of the BLCA datasets are shown in Tables 4 and 5. From the results of the four datasets in Table 4, it is obvious that the DCGN exhibits an excellent performance no matter which dataset is analysed. The DCGN performance is especially high for the BLCA-CIT-Curie and BLCA-TCGA datasets, and although the results of the other methods are also very good, the four indicators of DCGN at the highest level are nearly maximized, and the average results exceed 98%, indicating the best classification performance. Table 5 records the Kappa coefficient and Hamming distance values of all methods on the BLCA datasets. Table 5 shows that the DCGN has the highest Kappa coefficient and the smallest Hamming distance values on all datasets. On the TCGA and CIT-Curie datasets, the Kappa coefficient of the DCGN indicates that this was the only method to exceed 0.99, while the Hamming distances are only 0.005 and 0.006. In conclusion, the method proposed in this paper has a superior generalization ability and can learn different effective features to predict different classification tasks according to different datasets.

**Table 4:** Experimental results of BLCA datasets

Methods	DCNN	SVM	GBDT	LightGBM	gcForest	SAE	BiGRU	DCGN
Dataset	BLCA-MDA							
Accuracy	91.5 (90)	93 (91.1)	92.5 (91.4)	92.5 (90)	92.7 (89.5)	93 (92.1)	93.5 (92.7)	95.5 (94.2)
Precision	94.3 (92)	93.4 (91.2)	92.9 (90.6)	93 (90.4)	92.7 (90)	93.2 (92.2)	93.6 (92.6)	97.4 (94.5)
Recall	93 (91.8)	93.3 (91)	92.5 (90.4)	93.2 (90)	92.7 (90)	93 (92)	93.5 (92.4)	97.3 (94.2)
F1-score	93.3 (92)	93.4 (91)	92.6 (90.4)	93.3 (90)	92.6 (89.7)	93 (92.1)	93.4 (92.4)	97.3 (94.2)

Methods	DCNN	SVM	GBDT	LightGBM	gcForest	SAE	BiGRU	DCGN
Dataset	BLCA-Lund							
Accuracy	91.8 (90)	94 (91)	93.2 (90.2)	91.2 (90)	92.4 (90)	89.8 (88)	93.6 (92.5)	94.5 (93)
Precision	93 (91.5)	94 (91.2)	93.7 (91.8)	91.7 (90)	94 (91.5)	89.7 (88.1)	93.8 (92.7)	94.9 (93.7)
Recall	92.5 (90.7)	94 (91.1)	93.2 (90.4)	91.5 (89.6)	92.4 (90)	89.8 (88.4)	93.6 (92.5)	94.5 (93.4)
F1-score	92.6 (90.8)	94 (91)	92.9 (90.3)	91.6 (89.5)	92.6 (90)	89.6 (88.2)	93.7 (92.6)	94.5 (93.4)

Methods	DCNN	SVM	GBDT	LightGBM	gcForest	SAE	BiGRU	DCGN
Dataset	BLCA-TCGA							
Accuracy	93.3 (91.5)	98.3 (97)	98.4 (96.7)	98.5 (96.4)	98.5 (97.3)	95.4 (94.7)	97.4 (96.2)	99.3 (98.2)
Precision	97.3 (95.8)	98.5 (97.2)	98.3 (97)	98.6 (96.4)	98.6 (97.5)	95.7 (95)	97.4 (96.5)	99.4 (98.4)
Recall	96 (94.2)	98.4 (97)	98.3 (96.7)	98.4 (96.3)	98.6 (97.3)	95.4 (94.3)	97.3 (96.4)	99.3 (98.2)
F1-score	96.4 (94.8)	98.4 (97)	98.2 (96.8)	98.4 (96.5)	98.4 (97.4)	95.5 (94.6)	97.4 (96.4)	99.3 (98.2)

Methods	DCNN	SVM	GBDT	LightGBM	gcForest	SAE	BiGRU	DCGN
Dataset	BLCA-CIT-Curie							
Accuracy	96.1 (94.3)	98.5 (97)	98.2 (97.2)	97.8 (95.7)	98.3 (97.3)	98.3 (97.4)	97.8 (96.8)	99.4 (98.5)
Precision	98.5 (97)	98.5 (97.2)	98.3 (97.5)	98 (96)	98.4 (97.4)	98.4 (97.3)	97.9 (97)	99.5 (98.7)
Recall	98 (96.9)	98.4 (97.1)	98 (97)	97.8 (95.7)	98.2 (97.4)	98.2 (97.4)	97.6 (96.8)	99.4 (98.5)
F1-score	98.1 (96.8)	98.3 (97)	98.1 (97.2)	97.7 (95.6)	98.2 (97.3)	98 (97.3)	97.8 (96.9)	99.4 (98.5)

The value in front of () represents the highest-level result, and the value in () represents the average result over ten iterations

**Table 5:** Hamming distance and Kappa coefficient values obtained when applying models to BLCA datasets

	Kappa				Hamming distance			
Dataset	MDA	Lund	TCGA	CIT-Curie	MDA	Lund	TCGA	CIT-Curie
DCNN	0.896	0.908	0.956	0.978	0.067	0.081	0.024	0.02
SVM	0.91	0.93	0.98	0.984	0.059	0.06	0.019	0.014
GBDT	0.895	0.924	0.982	0.979	0.069	0.067	0.016	0.017
LightGBM	0.91	0.902	0.983	0.975	0.059	0.087	0.015	0.02
gcForest	0.881	0.915	0.983	0.98	0.079	0.075	0.015	0.016
SAE	0.895	0.898	0.941	0.98	0.069	0.096	0.045	0.016
BiGRU	0.903	0.929	0.966	0.973	0.064	0.063	0.021	0.022
DCGN	0.933	0.938	0.991	0.993	0.044	0.054	0.006	0.005

## Discussion

The method proposed herein still has some limitations. In this study, our method exhibits decent classification performances when applied to breast cancer and bladder cancer datasets. The subtype classifications of these cancers have been established; however, there are many cancers for which subtypes have not been accurately identified. Therefore, supervised learning of these other cancers is not yet possible. In addition, our approach to cancer classification is based solely on gene expression data. However, several recent studies have shown [35] that combining genomic data from different platforms can reveal more valid information about cancer subtypes; thus, this will characterize the next step of our research.

## Conclusions

In this paper, we proposed the DCGN, a deep learning method for cancer multiclassification tasks; this proposed model can better handle high-dimensional cancer data than other available models. In terms of classification evaluation indicators, such as the accuracy and precision, the DCGN performs well on all five analysed datasets, especially on the BLCA-TCGA and BLCA-CIT datasets, with values exceeding 99%. These results show that the method proposed in this paper can obtain ideal classification results and has a superior generalization ability. However, this paper considers only gene expression data, and we will integrate multiomics data to further study cancer subtype classification in future work.

## Supplementary Information

**Additional file 1:** Deep learning approach for cancer subtype classification using high-dimensional gene expression data.

## Data Availability

The BRCA unenhanced dataset can be obtained from http://www.acsu.buffalo.edu/~yijunsun/lab/DeepType.html. The BLCA unenhanced dataset is derived from the TCGA project. The software as part of this project is readily avail- able from GitHub at https://github.com/shijwe/DCGN

## Abbreviations

CNN: Convolutional neural network
BiGRU: Bidirectional gated recurrent unit
SVM: Support vector machines
ABC: Artificial bee colony algorithm
SAE: Stacked autoencoder
GBDT: Gradient boost decision tree
BRCA: Breast cancer susceptibility genes
BLCA: Bladder urothelial carcinoma
