# Supplementary material for: Deep learning approach for cancer subtype classification using high-dimensional gene expression data
Source: BMC Bioinformatics. 2022 Oct 17;23:430. doi: 10.1186/s12859-022-04980-9 (PMC9575247; doi:10.1186/s12859-022-04980-9)
Supplement: Supplementary file 1 — Additional file 1: Deep learning approach for cancer subtype classification using high-dimensional gene expression data. [file 12859_2022_4980_MOESM1_ESM.docx]

**Supplementary Materials**

Deep learning approach for cancer subtype classification using high-dimensional gene expression data

**S1 Comparative experiments of random undersampling and SMOTE algorithm**

Both random undersampling and SMOTE (upsampling) methods can equalize the data. So we conducted comparative experiments, and the experimental results are shown in Table S1 below.

As can be seen from the results in the table, after random undersampling, the classification performance of each method drops a lot, which is not ideal. Moreover, there is also an overfitting phenomenon on the BRCA and BLCA-Lund datasets. Therefore, the SMOTE algorithm may be more suitable for high-dimensional sparse datasets.

**Table S1.Performance of random undersampling and SMOTE algorithm on three datasets**

| Dataset | BRCA | | | | | | | | |
| --- | --- | --- | --- | --- | --- | --- | --- | --- | --- |
| Methods |  | RF | SVM | GBDT | LightGBM | gcForest | SAE | BiGRU | DCGN |
| Accuracy | SMOTE | 93.7 | 94.7 | 94.3 | 94.6 | 95.2 | 94.2 | 95 | **96** |
|  | UnderSampler | 80.9 | 80.6 | 81.7 | 81.7 | 82 | 82 | 81.7 | **82** |
| Precision | SMOTE | 93.8 | 94.9 | 94.5 | 94.7 | 95.4 | 95.3 | 95.4 | **98.7** |
|  | UnderSampler | 81.7 | 81.5 | 83.5 | 82.3 | 83.2 | 83.3 | 83 | **83.5** |
| Recall | SMOTE | 93.7 | 94.8 | 94.3 | 94.5 | 95.2 | 94.8 | 94.7 | **98.7** |
|  | UnderSampler | 80.8 | 81 | 81.7 | 81.7 | 81.8 | 82 | 81.8 | **82** |
| F1-score | SMOTE | 93.7 | 94.8 | 94.3 | 94.6 | 95.3 | 94.7 | 94.8 | **98.6** |
|  | UnderSampler | 80.9 | 81 | 81.9 | 81.6 | 81.8 | 82.1 | 82 | **82.3** |
| Dataset | BLCA-TCGA | | | | | | | | |
| Methods |  | RF | SVM | GBDT | LightGBM | gcForest | SAE | BiGRU | DCGN |
| Accuracy | SMOTE | 97.3 | 98.3 | 98.4 | 98.5 | 98.5 | 95.4 | 97.4 | **99.3** |
|  | UnderSampler | 91.7 | 90.4 | 92.8 | 92.7 | 93 | 92.8 | 92.9 | **93.1** |
| Precision | SMOTE | 97.4 | 98.5 | 98.3 | 98.6 | 98.6 | 95.7 | 97.4 | **99.4** |
|  | UnderSampler | 92.2 | 90.6 | 93 | 93.1 | 93 | 93 | 93.1 | **93.2** |
| Recall | SMOTE | 97.3 | 98.4 | 98.3 | 98.4 | 98.6 | 95.4 | 97.3 | **99.3** |
|  | UnderSampler | 92 | 90.4 | 92.8 | 92.8 | 92.8 | 92.5 | 92.6 | **92.8** |
| F1-score | SMOTE | 97.4 | 98.4 | 982 | 98.4 | 98.7 | 95.5 | 97.4 | **99.3** |
|  | UnderSampler | 92 | 90.2 | 92.7 | 92.6 | 92.7 | 92.6 | 92.7 | **92.7** |
| Dataset | BLCA-Lund | | | | | | | | |
| Methods |  | RF | SVM | GBDT | LightGBM | gcForest | SAE | BiGRU | DCGN |
| Accuracy | SMOTE | 92.4 | 94 | 93.2 | 91.2 | 92.4 | 89.8 | 93.6 | **94.5** |
|  | UnderSampler | 73.8 | 73.4 | 73.8 | 73.4 | 73.5 | 72.2 | 72 | **73** |
| Precision | SMOTE | 94 | 94 | 93.7 | 91.7 | 94 | 89.7 | 93.8 | **94.9** |
|  | UnderSampler | 76.5 | 76.4 | 76.3 | 78.4 | 77.5 | 78 | 75.6 | **88.4** |
| Recall | SMOTE | 92.4 | 94 | 93.2 | 91.5 | 92. | 89.8 | 93.6 | **94.5** |
|  | UnderSampler | 73.7 | 73.4 | 73.8 | 73.8 | 73.4 | 72 | 71.8 | **73** |
| F1-score | SMOTE | 92.5 | 94 | 92.9 | 91.6 | 92.6 | 89.6 | 93.7 | **94.3** |
|  | UnderSampler | 73.7 | 73.1 | 73.7 | 73.5 | 73.5 | 72.2 | 72.7 | **75** |

**S2 Comparative experiment of two convolutional layers**

The convolutional neural network contains two convolutional layers, conv1d and conv2d. We conducted comparative experiments on four datasets, and the experimental results are shown below.

**Table S2** **Performance of two convolutional layers on four datasets**

| BLCA-MDA | | | | |
| --- | --- | --- | --- | --- |
|  | Accuracy | Precision | Recall | F1-score |
| DCGN(conv1d) | 94.5 | 96 | 96 | 95.9 |
| DCGN(conv2d) | 95.5 | 97.4 | 97.3 | 97.3 |

| BLCA-Lund | | | | |
| --- | --- | --- | --- | --- |
|  | Accuracy | Precision | Recall | F1-score |
| DCGN(conv1d) | 93.2 | 95 | 94.5 | 94.3 |
| DCGN(conv2d) | 94.5 | 94.9 | 94.5 | 94.5 |

| BLCA-TCGA | | | | |
| --- | --- | --- | --- | --- |
|  | Accuracy | Precision | Recall | F1-score |
| DCGN(conv1d) | 99.3 | 99 | 99.2 | 99.1 |
| DCGN(conv2d) | 99.3 | 99.4 | 99.3 | 99.3 |

| BRCA | | | | |
| --- | --- | --- | --- | --- |
|  | Accuracy | Precision | Recall | F1-score |
| DCGN(conv1d) | 95.1 | 97.3 | 97.2 | 97.3 |
| DCGN(conv2d) | 96 | 98.8 | 98.7 | 98.7 |

As can be seen from Table S2, DCGN using conv2d performs well on the four datasets, and all four metrics are a bit higher than those of conv1d. Maybe the values of the two on a certain dataset are not much different, but in general, conv2d has better classification performance.


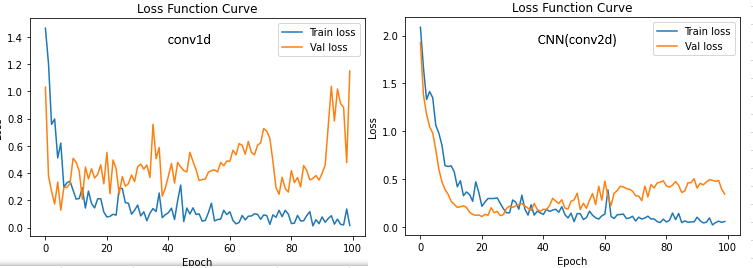


(a)


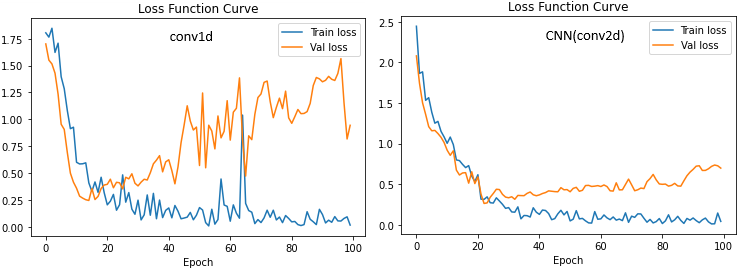


(b)


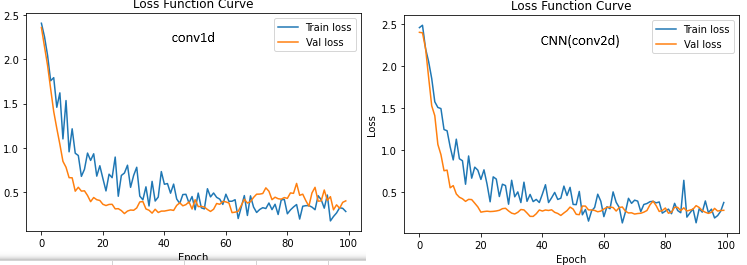


(c)


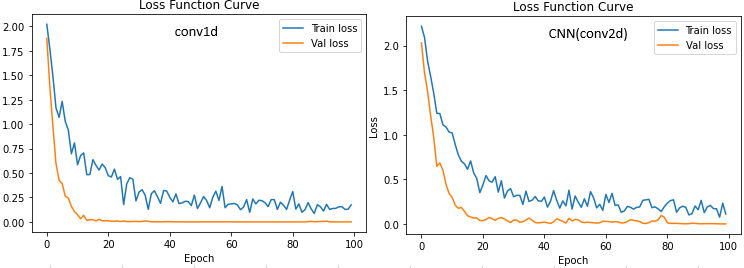


(d)

**Figure S1.Performance of two convolutional layers on four datasets. (a) represents the loss curve of two convolutional layers on the BRCA dataset; (b) represents the loss curve of two convolutional layers on the BLCA-MDA dataset; (c) represents the loss curve of two convolutional layers on the BLCA-Lund dataset; (d) represents the loss curve of two convolutional layers on the BLCA-TCGA dataset.**

Figure S1 are images of the loss curves of two convolutional layers on four datasets. On BRCA, the DCGN loss using conv2d is gradually flattened and relatively stable. On the BLCA-MDA dataset, conv1d has large fluctuations in the loss curve, and the loss does not converge, while conv2d is relatively stable and the loss gradually decreases. On both BLCA-TCGA and BLCA-Lund datasets, the loss curves of both convolutional layers are stable, and the loss values converge within a small range.

**S3 Comparative experiments of activation functions**

To verify the effect of different activation functions on model training, we selected four activation functions, gelu, relu, tanh and elu, to conduct comparative experiments on three datasets. The experimental results are shown in Figure S2.


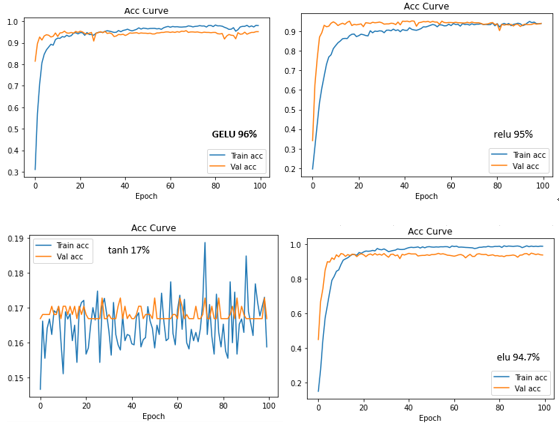


(a)


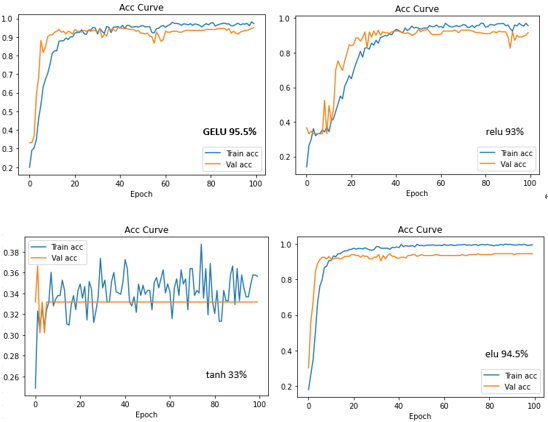


(b)


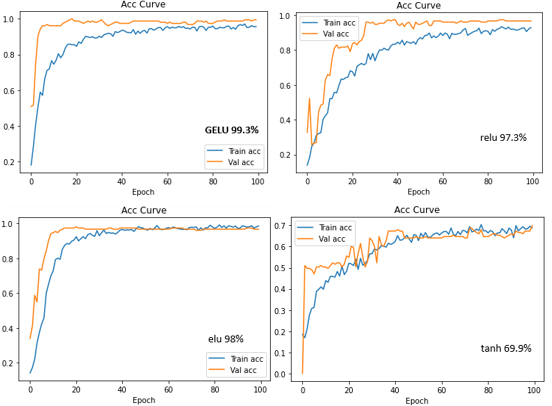


(c)

**Figure S2.Accuracy curves of different activation functions on three datasets. (a) represents the accuracy curve of the four activation functions on the BRCA dataset. (b) represents the accuracy curve of the four activation functions on the BLCA-MDA dataset. (c) represents the accuracy curve of the four activation functions on the BLCA-TCGA dataset.**

For the BRCA dataset, DCGN uses three activation functions: gelu, relu, and elu. The model convergence speed is very stable, but gelu has the highest accuracy. Not only is the accuracy of the tanh activation function low, but it does not converge after 100 epochs of training. On the BLCA-MDA dataset, it can be seen from the accuracy images that the training effect of DCGN using gelu and elu is very good, and it converges faster than relu, but the accuracy of gelu is still the highest. The effect of tanh is still very poor, and the accuracy of the verification machine finally stabilized at 33%. On the BLCA-TCGA dataset, it can be seen from the image that DCGN uses the gelu activation function to converge the fastest, and the accuracy rate is as high as 99.3%. Both relu and elu are a little less effective than gelu.

Based on the above results, we finally determined gelu as the activation function of the model.
